# Self-compassion and psychological well-being of radiographers at work

**DOI:** 10.1080/17482631.2023.2287621

**Published:** 2023-12-06

**Authors:** Leïla Jacquet, Marine Paucsik, Jean-Baptiste Guy, Karine Eve, Isabelle Ben-Taarit, Sophie Lantheaume

**Affiliations:** a Institut Supérieur Technologique Montplaisir, Valence (Drôme), France; b Univ. Grenoble Alpes, Univ. Savoie Mont Blance, Grenoble (Isère), France; c Centre Marie Curie, Valence (Drôme), France; d Ramsay Santé Hôpital Privé Drôme Ardèche, Guilherand-Granges (Ardèche), France

**Keywords:** Self-compassion, well-being, caregiver, radiographer, mixed study

## Abstract

**Objectives:**

The aim of this study was to assess how self-compassion affects the psychological well-being of radiographers at work.

**Methods:**

An online survey was sent to radiology and radiotherapy departments in Rhône-Alpes, a region of France (from October 2021 to February 2022). The study is mixed: quantitative data, with closed questions and two validated scales, and qualitative data, with open questions aimed at assessing perceptions among radiologists as regards self-compassion.

**Results:**

A total of 253 radiographers (mean age 32.9 years), took part in this survey. Radiographers reported a poor level of well-being and a moderate level of self-compassion. We found a link between well-being at work and self-compassion. Gender, age, number of years of experience and the desire to receive training on well-being appear to have an impact on the level of self-compassion. The perception of self-compassion by radiologists is essentially positive.

**Conclusion:**

Particular attention should be paid to radiologists who are female, young, and with only a few years of experience. Self-compassion is a protective factor for radiologists and may help them take care of themselves to continue caring for others. Training related to self-compassion should be promoted in medical imaging departments.

## Introduction

Psychological well-being is defined by a positive and balanced relationship with one’s work, with colleagues, and with oneself (Massé et al., 1998, 1998). There is a link between general well-being and well-being at work. According to Gilbert (Gilbert, 2009,) psychological health at work can be defined as “the capacity of individuals to satisfy their basic psychological needs in a perspective of betterment and adjustment to work, using personal and organizational resources.” In this context, psychological well-being at work does not arise from objective factors. It will hinge on the perceptions of each individual. In fact, work is an important part of a person’s life and individuals will spend almost one-third of their time at work (Abaidi, 2015). Work plays an important role in health and self-achievement (Debout et al., 2009,) and significantly contributes to building one’s identity (Lancry & Ponnelle, 2004). Several public health surveys in France have used work to study the health status of the population (Cohidon & Imbernon, 2009). Work is a source of stability and self-accomplishment, but also a source of physical and psychological vulnerability (Bressol, 2004; Davezies, 1999; Davezies et al., 2006).

Well-being at work, specifically among caregivers, can be influenced by the relationships that an individual entertains with his/her organization, colleagues, patients, work, and himself/herself (Chevalier et al., 2022). Among providers of care, several aspects of psychological well-being at work have been identified (Dagenais-Desmarais & Privé, 2010; Machado et al., 2016) including interpersonal fit, fulfilment, feeling competent, perceived recognition, and desire to engage. Radiographers are caregivers, but few studies have looked at the well-being of these professionals at work, whereas they are also affected by deteriorating working conditions. The nature of their work and the environment in which they operate may also be sources of stress (exposure to ionizing radiations and chemicals, accountability related to appropriate dosage, efficient organization of work, need for recognition, etc.) (Chevalier et al., 2022; Jasperse et al., 2014; Killion, 2009; Raj, 2006; Rajan, 2012; Shanahan & Akudjedu, 2021). Several studies showed that professional exhaustion among radiographers results from the fact that they are asked to carry out more tasks with fewer resources available, and to work in rotating teams due to staff shortages (Daugherty, 2002). The lack of breaks during the workday and high workload can also lead to stress and professional burnout (Eslick & Raj, 2000; 2002; Greenglass et al., 2001; Jasperse et al., 2014; Killion, 2009; Raj, 2006; Singh et al., 2017). Radiographers who worked for more than 10 hours per day reported higher levels of emotional exhaustion, depersonalization, and lower levels of personal accomplishment, compared to those who worked less hours (Singh et al., 2017). Excessive demands due to a lack of staff, to the pain experienced by patients, and even to aspects associated with a helper status, lead to overload and work-related exhaustion (Molinier, 2000). This adverse context exposes caregivers to professional burnout (Desrumaux, 2011; Desrumaux & Lemoine, 2012; Manoukian, 2009). Exhausted and disengaged, caregivers appear to be stripped of emotional resources to help them cope. The lack of social support, notably by colleagues, may also cause professional burnout among radiographers (Aronsson et al., 2017; Jasperse et al., 2014; Killion, 2009; Raj, 2006). Communication problems (contradictory demands, time constraints, lack of respect) are also reported in many studies as causes of suffering (Jasperse et al., 2014; Killion, 2009; Raj, 2006; Reingold, 2015). Finally, professional burnout may also be due to certain individual factors, such as a predisposition to emotional instability for example (Ghorpade et al., 2011; Yu et al., 2016). In addition, emotional vulnerability could also be due to problems with personal health, a family member who is ill, a death in the family, etc., all of which can make caregivers more fragile and less available to others.

One factor seems promising to support well-being: self-compassion (Zessin et al., 2015). Self-compassion has preventive and promotive strengths and allows to maintain harmony with internal and external aspects of life (Tiwari et al., 2020). Unlike self-esteem, self-compassion has no negative outcomes (Neff, 2011; Pandey et al., 2021). Self-compassion consists in letting oneself be affected by one’s own suffering, without trying to avoid or escape it, by trying to relieve it with kindness and understanding (Neff, 2003). More precisely, Neff (Neff, 2003) described three components of self-compassion: (1) kindness towards oneself. Self-compassion involves providing the same benevolence and kindness towards oneself that one would offer to another person. Unfortunately, while most individuals try to be benevolent and thoughtful towards their close ones when they are suffering (Germer & Neff, 2020,) they do not show similar attitudes towards themselves (Neff & Knox, 2017). If caregivers feel the same compassion towards themselves that they would to their best friend, they will react with warmth and understanding when faced with difficulties or failures. This benevolence requires the recognition that one is not perfect, and that it is inevitable and human to encounter difficulties. This will then reduce the frustration linked to the feeling of failure, and generate positive emotions of love and care, thereby helping them to cope (Germer & Neff, 2020). (2) Common humanity. The feeling of common humanity makes reference to a feeling of interconnection more than disconnection (Germer & Neff, 2020). To show self-compassion involves recognizing that suffering and difficulties are part of human experience (Germer, 2013). Common humanity also allows us to recognize that our thoughts, emotions, and behaviour are influenced by events outside our control. This helps to decrease the feeling of being alone, or injustice, linked to suffering or failure, to accept one’s weaknesses, and gives us the opportunity to feel close and more connected to others (Germer & Neff, 2020). (3) Mindfulness. Self-compassion also involves being aware of the emotions that we feel (Benzo et al., 2017; Raab et al., 2015; Sinclair et al., 2017). Showing compassion towards oneself means accepting a connection with suffering and tolerating its presence (Germer & Neff, 2020). Self-compassion involves a decentred attitude, allowing our experiences to be tackled internally in a different way. Boellinghaus, Jones, & Hutton (Boellinghaus et al., 2012) demonstrated that self-compassion reduces ruminations and therefore decreases symptoms of depression. Thus, self-compassion enables a person to accept that he/she is alive and to enjoy the present moment.

Available literature suggests that self-compassion is an interesting tool for carers (Montero-Marin et al., 2016; Raab, 2014; Vaillancourt & Wasylkiw, 2020). It is, among other things, associated with fewer symptoms of professional burnout. For example, nurses who have a high level of self-compassion are less likely to experience emotional exhaustion, to feel low levels of personal accomplishment, or to show depersonalization towards their patients (Vaillancourt & Wasylkiw, 2020). Self-compassion can also protect against post-traumatic stress (Beaumont et al., 2012; Thompson & Waltz, 2008) and has been associated with positive changes in the well-being of first-year student nurses, in part because they are more satisfied with their psychological needs (Gunnell et al., 2017).

Self-compassion also reduces the effect of stress on caregivers exposed to the suffering of patients and increases positive feelings at work. Kemper et al. (Kemper et al., 2015) reported that high levels of self-compassion indicate better resilience of practitioners and healthcare professionals. Furthermore, Allen & Leary (Allen & Leary, 2010) also demonstrated that self-compassion is a way of facing up to difficulties and stressful emotional experiences. Self-compassion is the opposite attitude to self-criticism, which is an important factor linked to anxiety and depression (Blatt, 1995). In other words, it protects against anxiety and depression (Neff, 2003; Neff et al., 2007). According to Stutts et al (Stutts et al., 2018,) 6 months of practice of self-compassion helps to prevent depression, anxiety, stress, and negative effects. Various studies have also demonstrated the protective role of self-compassion on suicidal ideation and self-harm (Jiang et al., 2016; Kelliher Rabon et al., 2018; Xavier et al., 2016). To have compassion for oneself appears to help carers take care of others (Hashem & Zeinoun, 2020). Individuals with self-compassion maintain a better emotional balance, enjoy a better daily life, and experience less chronic pain (Wren et al., 2012). Nurses with self-compassion who accept themselves as well as the experiences they live, by adopting a balanced emotional view of their work, are less likely to experience professional burnout and feel less dissatisfaction with regard to their work (Vaillancourt & Wasylkiw, 2020). Delaney (Delaney & Soundy, 2018) provided evidence of the advantages of self-compassion on the compassion and resilience of nurses and highlighted the importance of practicing self-compassion as a protective strategy for nurses.

A majority of previous studies have focused primarily on nurses. This exploratory study aims (i) to determine the link between the level of psychological well-being at work and the level of self-compassion in radiographers (i.e., the link between the five sub-dimensions of psychological well-being at work (interpersonal fit, exhaustion, feeling of competence, perceived recognition, desire to engage) and the six sub-dimensions of self-compassion (self-kindness, self-judgement, common humanity, isolation, mindfulness, and over-identification)); (ii) to determine the link between the level of well-being, the level of self-compassion, and sociodemographic variables (age, sex, type of workplace, type of training, etc.); (iii) to explore radiologists’perceptions of self-compassion.

Our results will help assess the relevance of proposing self-compassion as a tool in imaging services.

## Methods

### Study design

This exploratory study was conducted between October 2021 and February 2022 using an online questionnaire. We adopted a mixed method: qualitative and qualitative approaches were used simultaneously. The quantitative approach was based on closed questions and the use of two validated measurement scales. The qualitative approach, based on open questions, explored the perceptions of self-compassion in radiographers and assessed whether they had received any previous training on well-being. The mean duration for completion of the questionnaire was 15 min. Anonymization of respondents was achieved.

### Study participants

This study was targeted at all radiographers practicing in France. An email was sent to the managers of several medical imaging services in hospitals and private clinics in the Auvergne Rhône-Alpes region. The Association Française du Personnel Paramédical d’Électroradiologie (AFPPE) was contacted via LinkedIn and asked to share the study on its website. The study was also posted on several social media, with requests to participate (Facebook, Instagram).

The study participants were selected if they fulfilled the following inclusion criteria: (i) had obtained a diploma allowing them to work as a radiographer; (ii) were currently in post, in the private or public sector; (iii) worked with one of the following six methods of medical imaging procedure: computed tomography scans, magnetic resonance imaging, radiotherapy, interventional, nuclear medicine, ultrasound; and (iv) agreed to participate in the study.

### Tools

The questionnaire consisted of several parts: (i) an introduction with opening instructions; (ii) eleven closed questions; (iii) standardized scales; and (iv) two open questions.

The eleven closed questions, these made it possible to collect sociodemographic data.

The standardized scales are:

#### Psychological well-being

This was measured using the Index of Psychological Well-being at Work (IPWBW (Dagenais-Desmarais, 2010), which measures psychological well-being using five dimensions at work: (interpersonal fit, self-fulfilment, feeling of competency, perceived recognition, desire to engage). This tool consists of 25 items which are scored on a 6-point Likert scale with scores ranging from 0 (*Disagree*) to 5 (*Completely agree*). The closer a score is to 6, the higher the person’s level of well-being.

#### Self-compassion

The Self-compassion Scale (SCS (Neff, 2003) evaluates three components of self-compassion: (i) kindness towards oneself (versus self-judgement); (ii) common humanity (versus isolation); and (ii) mindfulness (versus over-identification). It consists of 26 items that are scored on a 6-point Likert scale with scores ranging from 1 (*Almost never*) to 5 (*Almost always*). The higher the score, the higher the person’s level of self-compassion.

Regarding the two **open questions**, respondents were invited to freely develop their perception of self-compassion and to investigate their real-life experiences in terms of training on well-being.

## Statistical analysis

Quantitative data collected from closed questions and standardized scales were analysed using *p*value.io (). Qualitative data collected from open questions were collated in order to carry out an analysis of thematic content (Bardin, 2009). This method consists in identifying recurring themes in verbal or textual material, based on key words, key expressions or salient phraseology (Mucchielli, 1996). It established the representation that radiographers had of self-compassion. The frequency of appearance of key terms is also presented to provide this qualitative analysis with more power.

## Results

### Quantitative data

In total, 253 radiographers, with a mean (SD) age of 32.9 ± 10.8 years (range: 20‒63 years), participated in this study. The majority of participants were female (84.2%). Table I summarizes the characteristics of the study population.

**Table I.** Characteristics of the study population.

Characteristic		
Sex	Male	40 (15.8)
	Female	213 (84.2)
Marital status	Single	70 (27.7)
	Married or civil partnership	106 (41.9)
	Cohabiting	70 (27.7)
	Separated or divorced	7 (2.8)
Family situation	No children	152 (60.1)
	With children	101 (39.9)
Structure in which the radiographer works	Public	15 (59.3)
	Private	99 (39.1)
	Both	4 (1.6)
Methodology used	Radiology	163 (64.4)
	CT scans	139 (54.9)
	MRI	71 (28.1)
	Radiotherapy	44 (17.4)
	Interventional radiology	22 (17.4)
	Nuclear medicine	22 (8.7)
Works as a manager	No	244 (96.4)
	Yes	9 (3.6)
Has already received training on well-being at work	No	231 (91.3)
	Yes	22 (8.7)
Would like to have training on well-being at work	No	42 (16.6)
	Yes	211 (83.4)
Age (years), mean ± SD		32.9 ± 10.8
No. of years’ experience, mean ± SD		10.5 ± 10.6
No. of children per radiographer, mean ± SD		2 ± 0.7

All results shown are n (%) unless stated otherwise.

CT: computed tomography; MRI: magnetic resonance imaging; SD: standard deviation.

#### Well-being of radiographers at work

The well-being of radiographers at work was poor (mean (SD): 3.88 ± 0.57), below normal levels on the scale used for this study. This was also true for all detailed aspects of well-being at work (Table II). There was a significant difference (1.72 points) between normal levels and that observed in the study population concerning “perceived recognition at work”.

**Table II.** Scores for the Index of perception of well-being at work (IPWBW) and self-compassion (SCS) scales.

IPWBW		3.88 ± 0.58
Sub-dimensions IPWBW	*Interpersonal fit at work*	4.11 ± 0.8
	*Exhaustion at work*	4 ± 0.76
	*Feeling of competence at work*	4.28 ± 0.62
	*Perceived recognition at work*	3.19 ± 0.93
	*Desire to engage at work*	3.84 ± 0.8
Self-compassion scale (SCS)		2.89 ± 0.55
Sub-dimensions of the SCS	Self-kindness	2.76 ± 0.78
	Self-judgement	2.74 ± 0.69
	Common humanity	3.04 ± 0.79
	Isolation	3.10 ± 0.88
	Mindfulness	3.12 ± 0.75
	Over-identification	2.60 ± 0.85

All values shown are mean ± standard deviation.

#### Self-compassion of radiographers

Participants reported a moderate level of self-compassion (mean (SD): 2.89 ± .55). This was the same for all detailed aspects of the SCS (Table II).

#### Well-being at work/Self-compassion of radiographers and sociodemographic variables

Statistical analyses did not demonstrate a link between well-being at work and sociodemographic variables.

**Figure 1.**
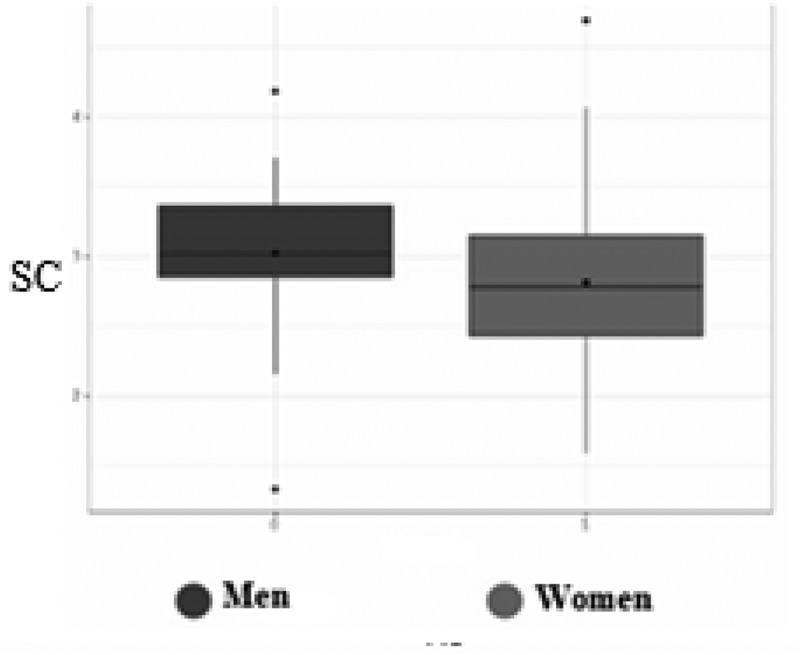
Difference in level of self-compassion (SC) according to gender of participants.

**Figure 2.**
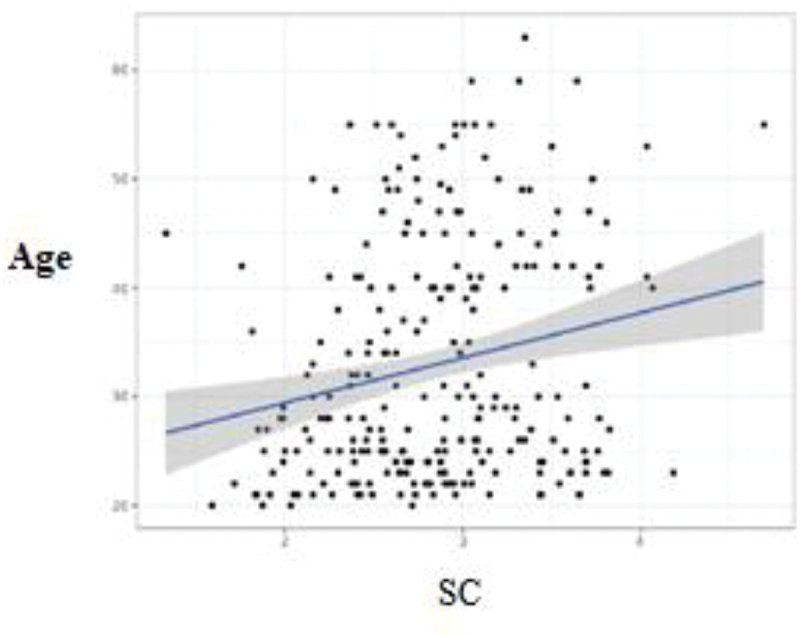
Correlation between self-compassion (SC) and participants’ age.

**Figure 3.**
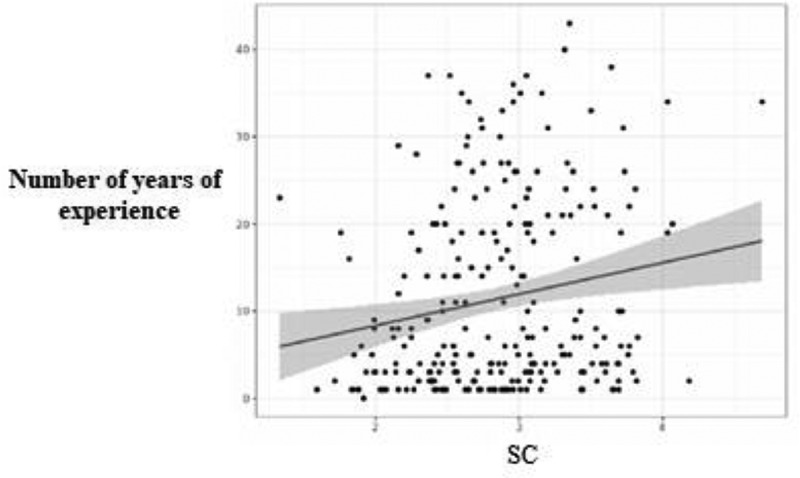
Correlation between self-compassion (SC) and the number of years of experience of the participants.

**Figure 4.**
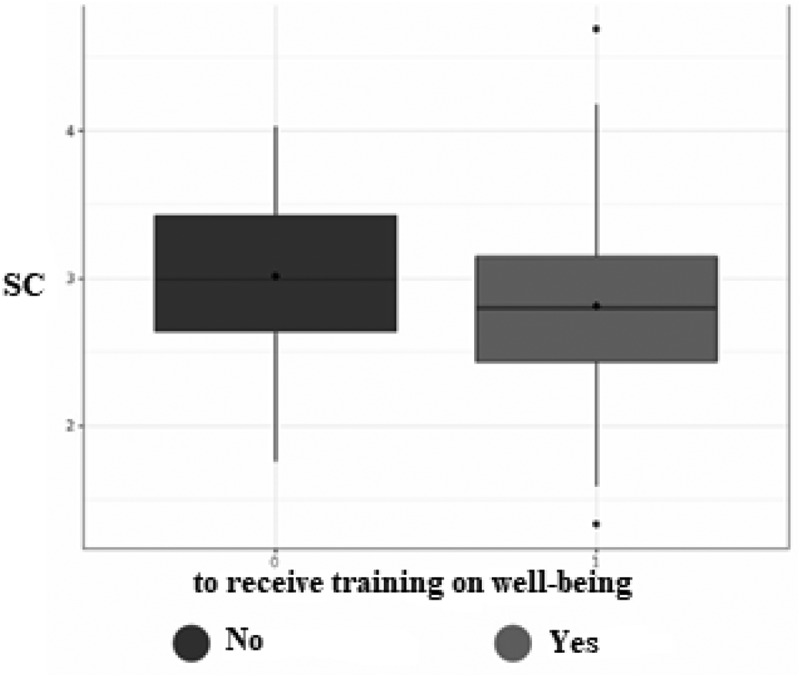
Difference in level of self-compassion (SC) according to the wish to receive or not a training on well-being.

Statistical analyzes demonstrated that there was a significant difference between male and female practitioners (*p* = .027). Men reported a higher level of self-compassion (mean (SD): 3.02 ± .54) than women (mean (SD): 2.81 ± .55) (Figure 1). There was also a statistically significant linear correlation between age and self-compassion (*r* = .21; *n* = 253; *p* < .001): the older the radiographer, the higher the level of self-compassion (Figure 2). There was a statistically significant linear correlation between the number of years of experience as a radiographer and self-compassion (*r* = .18; *n* = 253; *p* < .01): the more years of experience, the higher the level of self-compassion (Figure 3). Finally, there was a significant difference between the radiographers who wished to receive training on well-being and those who did not (*p* = .026): the radiographers who reported a high level of self-compassion did not want to receive training on well-being (mean (SD): 3.02 ± .52), in contrast to radiographers who reported a low level of self-compassion who wanted to receive training on well-being (mean (SD): 2.81 ± .55) (Figure 4).

#### Well-being at work and self-compassion of radiographers

**Figure 5.**
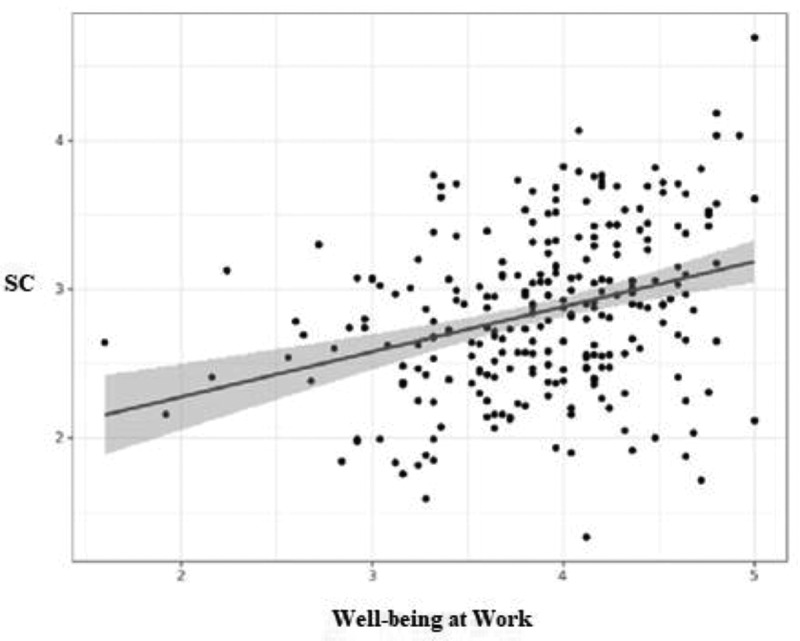
Correlation between self-compassion (SC) and well-being at work.

Statistical analysis revealed a significant linear correlation between self-compassion and IPWBW (*r* = .32; *n* = 253; *p* < .001): the higher the level of self-compassion, the higher the IPWBW (*p* < .001) (Figure 5).

#### Training

At the time of the study, no radiographer who responded to the study had received training on self-compassion. The main types of training reported were in relation to stress management (48.3%), positive psychology (13.8%), the management of emotions (10.3%), and to working as part of a team (10.3%). Table III shows the training previously received by the respondents.

**Table III.** Training received by the participants prior to the study.

Theme	n (%)	*Citations*
Management of stress	14 (48.3)	*Management of stress* *Relaxation* *Hypnosis* *Mindfulness meditation* *Reiki* *Self-hypnosis*
Positive psychology	4 (13.8)	*Positive psychology* *Compassion* *Take care of themself by taking care of others* *Carer/patient relationship*
Management of emotions	3 (10.3)	*Emotional intelligence* *Management of emotions*
Working as part of a team	3 (10.3)	*Management of conflict* *Non-violent communication Team cohesion*
Touch	2 (6.9)	*Taking care by touch and relaxation*
Personal development	2 (6.9)	*Personal development* *Coaching*
Other	1 (3.5)	*Kundalini yoga*

## Qualitative data

### Perception of self-compassion

In total, 722 words were cited in response to the open question on radiologists’ perception of self-compassion, including 162 different ideas.

These terms could be grouped into three categories: 122 positive ideas (75.3%), 18 negative ideas (11.1%), and 22 neutral ideas (13.6%).

The positive word that appear most often was “*kindness*” (cited 71 times by 46.4% of participants), followed by the words “*listen*” (cited 44 times by 28.8%), “*acceptance*” (cited 41 times by 26.8%), “*understanding*” (cited 41 times by 26.8%), “*tolerance*” (cited 38 times by 24.8%), and “*empathy*” (cited 36 times by 23.5%). Among the words with a negative connotation, the terms most frequently cited were “*compassion*” (cited three times by 2.0% of the participants), “*self-judgement*” (cited three times by 2.0%) and “*pity*” (cited twice by 1.3%). As for neutral words, the terms “*support*”, “*education*”, and “*feeling*” were used.

By classifying the terms reported according to the three categories which correspond to the detailed aspects of self-compassion, “*kindness towards oneself*” was cited on 278 occasions (38.5%), while “*common humanity*” was evoked on 255 occasions (35.3%), and “*mindfulness*” on 66 occasions (9.1%).

There was a net difference between the three dimensions of self-compassion, indicating a disparity in knowledge and representation.

## Discussion

The aim of this study was to evaluate the link between levels of psychological well-being at work and levels of self-compassion, the link between levels of well-being, levels of self-compassion and sociodemographic variables (type of workplace, type of training, etc.), and to investigate the representations of self-compassion among radiographers.

The results show that levels of well-being at work of radiographers are poor and level of self-compassion moderate. There is a link between well-being at work and self-compassion. Gender, age, number of years of experience and the desire to have training on well-being have an effect on the level of self-compassion. Finally, the perception of self-compassion by radiologists is essentially positive.

Well-being at work is lower than the norm, including all sub-dimensions of well-being at work. Our results confirm those of other studies on burnout (Jasperse et al., 2014; McHugh et al., 2011; Sale & Smoke, 2007; Singh et al., 2017; Sipos et al., 2019) which show high levels of professional exhaustion among carers.

In addition, there was a significant difference in the score obtained and normal levels for the item “perceived recognition at work”, one of the dimensions of psychological well-being at work (Dagenais-Desmarais, 2010). This observation confirms those of several other studies (Aronsson et al., 2017; Jasperse et al., 2014; Killion, 2009; Raj, 2006) which show the lack of support from colleagues and a lack of recognition of radiographers. This lack of recognition was also demonstrated in the report of France’s General Inspectorate of Social Affairs (GISA) () of February 2021. This dimension therefore appears to play a negative role in the well-being of radiographers at work. Furthermore, interpersonal fit at work also registered scores below the norm, demonstrating that radiographers are not satisfied with their relationships with the people with whom they interact in the context of their work. This element therefore demonstrates that interpersonal problems can be added to the lack of recognition. It is therefore appropriate to improve the Index of Psychological Well-being at Work of radiographers, as highlighted in the GISA report, to improve the recognition of radiographers using concrete methods such as higher salaries, better recognition of practitioners, protocols for better cooperation between colleagues, and more generally any practice used to combat the lack of understanding of a given profession by the general public.

The dimension that achieved the highest score was the feeling of competence at work, which reflects the feeling of personal accomplishment, another of the components of well-being at work (Ryan & Deci, 2000). Satisfaction in this area has a positive effect on the well-being of workers (Van den Broeck et al., 2008). It can be assumed that the feeling of competence plays an important role in the IPWBW of radiographers, even though this remained below the norm on the scale. It should also be noted that the dimensions “commitment to work” and “exhaustion at work” were also below the norm, making each of them areas for improvement to increase the well-being of radiographers at work (Dagenais-Desmarais & Privé, 2010,) to better deal with emotions and negative thoughts, and to develop greater engagement with work (Morgan et al., 2015).

The results of our survey demonstrate moderate levels of self-compassion among radiographers, which was also true for all of the dimensions of self-compassion (kindness to oneself, common humanity, and mindfulness). These results are discussed in the light of studies carried out among nurses, because no study to date has investigated self-compassion among radiographers.

The participants in our study had lower levels for all dimensions of self-compassion than the nurses in the studies of Vaillancourt & Wasylkiw (Vaillancourt & Wasylkiw, 2020) and Heffernan et al. (Heffernan et al., 2010) except for the dimension “over-identification”. The most obvious difference was for the dimension “mindfulness”. However, it was noticed that caregivers in these two studies had moderate levels of self-compassion. This can be explained by the fact that caregivers had difficulty connecting with their emotions because of the representations of their profession, probably to hide their emotions for fear of appearing weak, vulnerable, or to safeguard patients for example.

Our results also show that self-compassion is significantly linked to the levels of well-being. As demonstrated in some studies, self-compassion facilitates adaptation (Allen & Leary, 2010; Jenaro et al., 2007; Leary et al., 2007). In addition, self-compassion allows an individual to better face up to tensions at work and to develop better well-being. This may explain why a person showing self-compassion, who is sliding towards burnout, can cope by using the principles of self-compassion. By trying to stand back from negative situations, by adopting a compassionate attitude towards himself/herself. This confirms other studies which have shown the positive effect of self-compassion on burnout (Duarte et al., 2016; Durkin et al., 2016; Montero-Marin et al., 2016; Olson et al., 2015). In the study of Vaillancourt & Wasylkiw (Vaillancourt & Wasylkiw, 2020,) the nurses who had a high level of self-compassion were less likely to experience emotional exhaustion, which is linked to satisfaction and the quality of sleep. In addition, another study demonstrated that self-compassion could act as a moderating factor between professional burnout and well-being (Kyeong, 2013) in caregivers (Hashem & Zeinoun, 2020). Self-compassion therefore seems to be a protective factor for radiographers and appears to help them take care of others.

Our results show that male radiographers had a higher level of self-compassion than female radiographers, which is similar to the conclusions of the study by Yarnell et al (Yarnell et al., 2015). This result can be explained by the fact that women may tend to be more self-critical and to ruminate more than men (Leadbeater et al., 1999; Nolen-Hoeksema et al., 1999). We also observed a relation between levels of self-compassion and age as well as with the number of years of experience. The older the radiographers, the greater their levels of self-compassion. This corroborates previous studies which reported a positive correlation between self-compassion and age (Hwang et al., 2016; Neff & Vonk, 2009; Wren et al., 2012). This can be explained by self-criticism which has a tendency to decrease with age. As people age, they have a tendency to see a more positive image of themselves, because of the experience that they have acquired in their professional and personal life (Kopala-Sibley et al., 2013). These results show the same trend as the link between level of experience and self-compassion. In effect, as the radiographer gets older, the more their level of experience increases and the more their level of self-compassion also increases.

Concerning the perception of self-compassion among radiographers, our results show a positive representation of this concept, even though some caregivers reported words with a negative connotation such as “self-pity” or “self-judgement”. The radiographers expressed the wish to obtain information on self-compassion. When they thought of self-compassion, the three dimensions of self-compassion were not evoked directly. For most of the radiographers, the term self-compassion referred to kindness, a bit less to mindfulness, but few had thought of the idea of common humanity. We can suppose that any proposal of training on self-compassion would be received positively by the participants.

Radiographers requested training on well-being and none had ever followed a program dedicated to self-compassion. We were surprised to observe that the radiographers with a high level of self-compassion were less likely to express the wish to receive training on well-being than those with a lower level of self-compassion. Self-compassion requires a person to be aware of his/her emotions and needs, and to be able to take a step back from who they are and what they should do. In addition, the level of well-being among the radiographers observed was quite low, where we would have expected the opposite relationship: the more the level of self-compassion increases, the higher the desire to have training on well-being increases. Self-compassion requires a benevolent attitude, understanding and attention towards oneself. However, studies show that self-compassion is not correlated with the acceptance of receiving compassion from other people (Dupasquier et al., 2018). People showing self-compassion are not able to accept external help to take care of themselves. We know that the thought of accepting compassion from another person is negatively correlated with mental health (Kirby et al., 2019). This could therefore explain why individuals with moderate levels of self-compassion also have low levels of well-being. Future studies could explore these dimensions by using the Compassionate Engagement and Action Scale (CEAS (Gilbert et al., 2017) or the Fear of Compassion Scale (Gilbert et al., 2011).

A significant number of studies have shown that caregivers can learn self-compassion. Shapiro et al. (Shapiro et al., 2005) reported that people trained in mindfulness increase their capacity for self-compassion, which leads to a decrease in their levels of stress. In addition, therapists who participate in online training on self-compassion have, upon completion of a 6-week program, decreased their stress, enjoy better control of their emotions, and have increased their well-being (Finlay-Jones et al., 2015). There are initiatives on mindfulness training which aim to reduce stress among healthcare professionals (Aycock & Boyle, 2009; Zeller & Levin, 2013). Training programs, such as the Mindful Self-Compassion Program (MSC (Neff, 2012), Compassion Cultivation Training (CCT™ (Goldin & Jazaieri, 2017), or the Compassion Focused Program (Paucsik et al., 0000,) have also been developed to improve self-compassion (Zessin et al., 2015). Some researchers (Kristeller & Johnson, 2005) have declared that the practice of meditation is a good way to improve the qualities of self-compassion and empathy (Newsome et al., 2012). A recent study by Brun et al. (Brun et al., 2021) concluded that professional training programs on mindfulness (MBSR) (Kabat-Zinn, 2003a, 2003b) are a good means for developing self-compassion in caregivers. These training programs are excellent tools for institutions: they support an empathetic relationship between the carer and their patients and help improve the efficacy of care (Brun et al., 2021).

One of the main strengths of this study is that it investigates a novel area of research (well-being at work and self-compassion among radiographers) which has received little attention in previous studies. It shows real suffering among radiographers at work, the need for training, and addresses the prevention of professional burnout. Methodologically, the collection of both quantitative and qualitative data provides useful information.

This study also has some limitations. The first limitation concerns the representativeness of the study population. Our population contained more women than men, and furthermore, professional affiliations with the private and public sectors were not balanced in the sample. In addition, our questionnaire did not reveal the geographic origin of the radiographers questioned. Methodologically, as in many studies that used an online self-questionnaire, this could be the source of bias (Lindell & Whitney, 2001; Podsakoff et al., 2003). This means that the correlations between self-compassion and well-being at work could include an error of measurement resulting from the methods by which the survey was administered. We can hypothesize that the radiographers who responded to the questionnaire were perhaps those who experienced the most suffering at work. In effect, if they experienced less suffering, they may have experienced less need to express themselves and the subject was perhaps less interesting to the radiographers who felt comfortable with their professional tasks. Furthermore, details on the radiographers’ working conditions were not collected. Finally, it would be interesting to continue the study with a controlled, randomized trial, by proposing training on self-compassion to radiographers and to compare the long-term effects on well-being at work.

## Conclusion

This study reveals the need to improve the well-being at work of radiologists and to pay particular attention to radiologists who are female, young, and with a low number of years of experience. This improvement can be achieved through higher recognition of the profession (notably by fighting against ignorance of the profession among the general public) but also through concrete actions (increased salaries, development of cooperation protocols between colleagues, etc.). Self-compassion appears to be a protective factor for radiologists that would help them take care of themselves to continue caring for others. Providing training on self-compassion to radiologists would increase their well-being at work but also help them develop skills to overcome work organization problems and prevent professional burnout.

## Supplementary Material

Short biographical note for each author.docx
