# Supplementary material for: Self-compassion and psychological well-being of radiographers at work
Source: Int J Qual Stud Health Well-being. 2023 Dec 6;19(1):2287621. doi: 10.1080/17482631.2023.2287621 (PMC11737828; doi:10.1080/17482631.2023.2287621)
Supplement: Short biographical note for each author.docx [file ZQHW_A_2287621_SM7157.docx]

**Short biographical note for each author**

**Leïla Jacquet** is Radiographer. She completed her final dissertation in medical imaging on self-compassion among radiographers. His interests are centered on the welfare of caregivers. She wants to use the results of this study within her department by developing a program centered on self-compassion to improve the quality of life of caregivers in her department.

**Marine Paucsik (McF)** is Psychologist and McF at University of Savoy and Associate Researcher at LIP/PC2S. She has developped an expertise in self-compassion over the years. She is the author of several books and numerous articles on the subject.

**Jean-Baptiste Guy (PhD)** is Radiotherapist and Oncologist at Centre Marie Curie. He takes a major interest in the well-being of his employees and participates in the development of actions within his center in favor of the well-being onf the healthcare teams.

**Karine Eve** : Radiographer and head teacher at Institut Supérieur Technologique Monplaisir. She supports studies addressing the well-being of future caregivers.

**Isabelle Ben-Taarit (PhD)** is Radiologist at Hôpital Privé Drôme Ardèche. Invested within her teams, she develops projects to improve the well-being of caregivers.

**Sophie Lantheaume (PhD)**, in a Psychologist, Supportive Care team Manager, Clinical research, oncology coordinator, at Ramsay Santé Hôpital Privé Drôme Ardèche and Associate Researcher at LIP/PC2S University of Grenoble Alpes. She is the author of several books and her work focuses on improving the quality of life of caregivers and patients.

Other information includes on ORCID **:** [**https://orcid.org/0000-0001-9864-4987**](https://orcid.org/0000-0001-9864-4987)
